# Long-term outcomes following posterior fossa decompression in pediatric patients with Chiari malformation type 1, a population-based cohort study

**DOI:** 10.1007/s00701-024-06332-3

**Published:** 2024-11-18

**Authors:** Victor Gabriel El-Hajj, Erik Öhlén, Ulrika Sandvik, Jenny Pettersson-Segerlind, Elias Atallah, Pascal Jabbour, Mohamad Bydon, David J. Daniels, Adrian Elmi-Terander, Erik Edström

**Affiliations:** 1 Department of Clinical Neuroscience, Karolinska Institutet, Stockholm, Sweden; 2 Department of Neurological Surgery, Thomas Jefferson University Hospital, Philadelphia, PA USA; 3 Department of Neurosurgery, Mayo Clinic, Rochester, MN USA; 4 Capio Spine Center Stockholm, Löwenströmska Hospital, 194 02, Box 2074, Upplands-Väsby, Sweden

**Keywords:** Chiari malformation type 1, Pediatric, Posterior fossa decompression, Suboccipital decompression, Foramen magnum decompression

## Abstract

**Objective:**

Posterior fossa decompression for Chiari malformation type I (Chiari 1) is effective and associated with a low risk of complication. However, up to 20% of patients may experience continued deficits or recurring symptoms after surgical intervention. For pediatric patients, there are no established tools to predict outcomes, and the risk factors for unfavorable postoperative outcomes are poorly understood. Hence, our aim was to investigate baseline data and early postoperative predictors of poor outcomes as determined by the Chicago Chiari outcome scale (CCOS).

**Methods:**

All pediatric patients (< 18 years) receiving a posterior fossa decompression for Chiari 1 between the years of 2005 and 2020 at the study center were eligible for inclusion. Patients with congenital anomalies were excluded.

**Results:**

Seventy-one pediatric patients with a median age of 9 years were included. Most patients (58%) were females. Chiari 1 was associated with syringomyelia (51%), scoliosis (37%), and hydrocephalus (7%). Perioperative complications occurred in 13 patients (18%) of which two required additional procedures under general anesthesia. On multivariable proportional odds logistic regression, motor deficits (OR: 0.09; CI95%: [0.01–0.62]; *p* = 0.015), and surgical complications (OR: 0.16; CI95%: [0.41–0.66]; *p* = 0.011) were significant predictors of worse outcomes. The presence of syringomyelia was identified as a predictor of better outcomes (OR: 4.42 CI95% [1.02–19.35]; *p* = 0.048). A persistent hydrocephalus during the early postoperative period after posterior fossa decompression was a strong predictor of worse long-term CCOS (OR: 0.026; CI95%: [0.002–0.328]; *p* = 0.005).

**Conclusion:**

Results from this study indicate that the existence of motor deficits and syringomyelia prior to surgery, and surgical complications and persistent hydrocephalus despite posterior fossa decompression, were useful predictors of long-term outcome.

## Introduction

Chiari Type I Malformation (Chiari 1) is a congenital neurological disorder that is thought to result from the interaction of several factors, of which embryonic underdevelopment of the occiput is the most critical [9]. The pathology is characterized by a smaller and shallower posterior cranial fossa [47], especially in relation to the cerebellar volume [40, 50]. Overcrowding in the posterior fossa results in tonsillar descent through the foramen magnum, tonsillar ectopia, and CNS structures within the foramen magnum may be compressed. In the pediatric population, Chiari 1 often coexists with syringomyelia, hydrocephalus, scoliosis, craniosynostosis, or tethered spinal cord [42]. It is estimated that 1 in 1000 individuals have a clinically overt condition, however, radiological signs of Chiari 1 may incidentally be found in up to 1% of the general population [3, 11, 39, 51]. The symptoms and clinical features of Chiari 1 fall under a wide spectrum ranging from the classical exertional headache, to neck pain, sensory or motor deficits, dysphagia, sleep apnea, or even developmental, psychologic and cognitive impairment in younger patients [35, 44]. Posterior fossa decompression, the treatment of choice in the management of Chiari 1, is often reserved to symptomatic patients, or those with concomitant syringomyelia or scoliosis [4, 25, 30, 41]. Surgical treatment is effective and associated with a low risk of complications, with around 75% of patients experiencing favorable outcomes and postoperative improvements [5, 6, 18, 33, 34]. However, up to 20% of patients may experience continued deficits or recurring symptoms after surgical intervention [5, 6, 10]. In the adult population, there have been repeated efforts to develop outcome predictors for posterior fossa decompression in Chiari 1 [33]. However, for pediatric patients, there are no such tools to predict outcomes, and the risk factors for unfavorable postoperative outcomes are poorly investigated. The aim of this study was to review the institutional experience with posterior fossa decompression for children with Chiari 1, to describe the long-term postoperative clinical and radiological outcomes and identify possible outcome predictors.

## Methods

### Study center and patient selection

The study hospital is a publicly funded and owned tertiary care center serving a region of roughly 2.3 million inhabitants, and the only neurosurgical center in the region. All pediatric patients (< 18 years) receiving a posterior fossa decompression between the years of 2005 and 2020 at the study center were screened for inclusion in this study. Exclusion criteria were: a posterior fossa decompression for causes other than Chiari 1; Chiari 2, a concomitant congenital syndrome or deformity, including craniosynostosis, achondroplasia, Noonan’s and Crouzon’s syndromes; incomplete or unavailable medical records.

### Study design and ethical considerations

The study is a retrospective population-based cohort study of patients with Chiari 1 that were consecutively treated with posterior fossa decompression at the authors’ institution. This study was conducted according to the Declaration of Helsinki and approved by the Swedish Ethical Review Authority (Dnr: 2018 − 1873–31, 2024-03244-01). Due to the retrospective nature of the study and the anonymized dataset used, the ethical review board waived the need for informed consent. The study conforms to the RECORD reporting guidelines (Supplementary file 1).

### Variables

An electronic patient chart review performed on the health record software TakeCare (CompuGroup Medical Sweden AB, Farsta, Sweden) was conducted to retrieve data on the included patients. Baseline patient characteristics included biological sex, age at time of diagnosis, BMI, presenting symptoms and their duration. All patients were radiologically evaluated preoperatively. Imaging data was collected regarding the cerebellar tonsillar descent (CTD) grade, syringomyelia, hydrocephalus, scoliosis, platybasia, and basilar invagination. The CTD was graded as previously described [53], grade 1: tonsils descend through the foramen magnum without reaching the C1 arch - grade 2, tonsils descend to the C1 arch level - grade 3, tonsils descend below the C1 arch. Platybasia was defined as a basal angle > 143 degrees as measured by standard techniques [32], while basilar invagination was defined as an abnormal protrusion of the odontoid into the foramen magnum [46]. The Chiari severity index (CSI) was calculated for all patients [20]. Perioperative complications were reported using the Ibanez grading scheme [27]. An assessment of functional and neurologic status was performed for every patient preoperatively, early postoperatively and at last follow-up (median: 8 years). The American society of anesthesiologist (ASA) score and the modified McCormick score (mMCs) were used. The Chicago Chiari Outcome Scale, CCOS, a validated Chiari 1 specific clinical outcome score, was used to assess the degree of postoperative improvement. Radiologic outcomes were evaluated at early (median 5 months) and final follow-ups (median 5 years).

### Description of the surgical procedure

The surgical procedure was performed on a prone patient with the head positioned to allow suboccipital access in a mayfield clamp or horseshoe head rest depending on the age of the patient. Through a midline incision, a suboccipital craniectomy of 3 × 3 cm and a 3-cm wide laminectomy of C1 were conducted using an ultrasonic bone scalpel (Misonix Inc.) or a high-speed drill with a diamond-coated burr. The dimensions of the bony opening were adjusted to compensate for the smaller dimensions of the younger patients. The atlantooccipital membrane and the constrictive band under C1 were removed. Under the microscope, the dura was incised in a Y-shaped fashion and held open by sutures, allowing exposure of the cerebellar tonsils. These sutures were kept at closing and allowed for tenting of the duraplasty. The arachnoid was dissected sharply, and the cerebellar tonsils untethered. Adhesions obstructing the passage of CSF at the obex were removed. A dura graft (Lyoplant, B Brown) was adapted to the size of the dural opening and sutured, watertight, with resorbable sutures. Fibrin glue (Tisseel, Baxter or Evicel, Omrix) was applied, and an additional dura substitute (DuraGen, Integra) was placed over the sutured duraplasty. The soft tissues were then sutured in layers to close the wound. Skin staples were used and removed after 10 days.

### Statistics

Medians and interquartile ranges (IQR) are used to describe continuous data while categorical data are presented using numbers and proportions. The Chi-squared, and when appropriate, the Fisher’s Exact tests were used to compare categorical data across groups, while the Mann-Whitney U test was used for the comparison of continuous data across binary groups. The Wilcoxon signed rank and McNemar tests were used to determine the significance of changes in mMC, and CCOS at different time points. A proportional odds logistic regression model was used to determine predictors of long-term CCOS using either pre- or perioperative clinical parameters or early imaging outcomes. All statistical analyses were performed in the SPSS statistics software, version 26, and p-values of < 0.05 were considered significant.

## Results

### Baseline patient characteristics and radiological findings

**Fig. 1:**
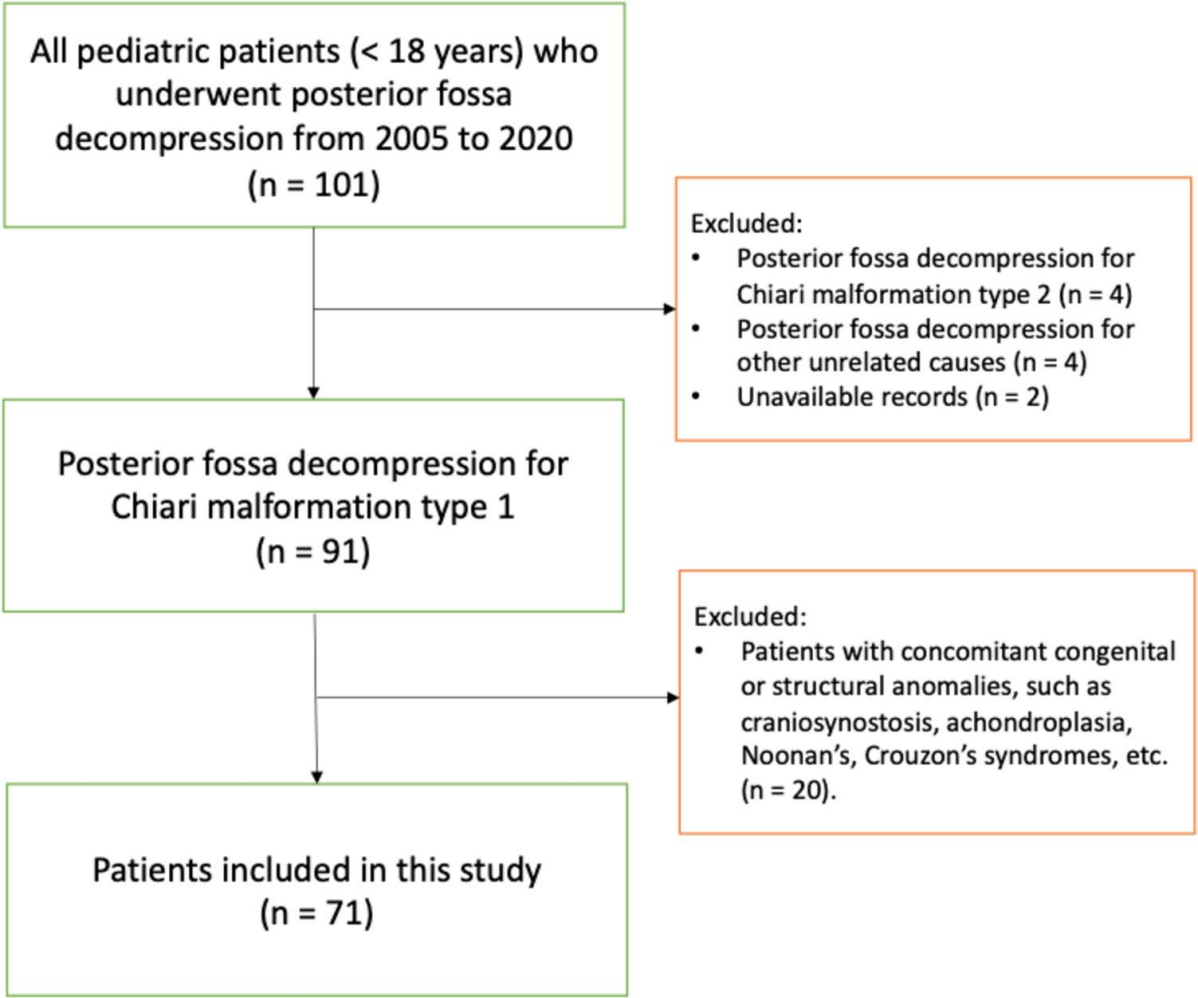
Patient selection flowchart

Following the selection process, 71 pediatric patients (< 18 years) were included in the study (Fig. 1). The overall cohort was characterized by a predilection of females (58%), a median age of 9 (IQR: 5–13), and BMI of 17 (IQR: 15–19). The most common presenting symptoms were headaches (58%) and vertigo or impaired balance (25%). The duration of symptoms before diagnosis extended to a median of 11 months (IQR: 1–21; Table 1).

**Table 1:** Patient characteristics, surgical management and complications

Baseline characteristics	
Female sex	41 (58%)
Age	9 (5–13)
BMI (1 missing)	17 (15–19)
**Presenting signs and** **symptoms**	
Headache	41 (58%)
Vertigo or impaired balance	18 (25%)
Nausea/vomiting	13 (18%)
Neck pain	12 (17%)
Sensory disturbances	12 (17%)
Motor deficit	10 (14%)
Sleep apnea	6 (9%)
Dysphagia	2 (3%)
Symptom duration (mos) (21 missing)	11 (20)
**Imaging findings**	
Cerebellar Tonsil Descent (CTD)	
Grade 1 (below FM)	13 (18%)
Grade 2 (at C1 arch)	29 (41%)
Grade 3 (below C1 arch)	29 (41%)
Syringomyelia	36 (51%)
Syrinx location	
Cervical	7/36 (19%)
Cervical to thoracic	17/36 (47%)
Thoracic	3/36 (8%)
Thoracal to lumbar	1/36 (3%)
Cervical to lumbar	8/36 (2%)
Syrinx diameter ≥ 6 mm	22/36 (61%)
Hydrocephalus	5 (7%)
Platybasia	1 (1%)
Basilar invagination	8 (11%)
Scoliosis	26 (37%)
**Presenting clinical scores**	
Modified McCormic (mMC)	1 (1–1)
Chiari Severity Index (CSI)	
1	25 (35%)
2	26 (37%)
3	20 (28%)
**Posterior fossa decompression surgery**	
Dura graft placement (duraplasty)	68 (96%)
Tonsillar cauterization	37 (52%)
**Complications (Ibanez grade)**	13 (18%)
1a (spontaneously resolving)	3/13 (23%)
1b (requiring pharmacological treatment)	6/13 (46%)
2a (requiring invasive treatment without general anesthesia)	2/13 (15%)
2b (requiring invasive treatment with general anesthesia)	2/13 (15%)
Length of hospital stay (days)	6 (5–8)
Reoperation	9 (13%)

Abbreviations: CTD = cerebellar tonsillar descent, mMC = modified McCormick Scale, CSI = Chiari severity index

On preoperative imaging, the CTD grade was 1 in 18%, 2 in 41%, and 3 in 41%. Syringomyelia was seen in 51% of the patients and most commonly extended from the cervical to the thoracal regions (47%). In 61% the diameter of the syrinx exceeded 6 mm. Scoliosis was present in 37% (*n* = 26), and hydrocephalus in 7% (*n* = 5) of the patients. Radiographic measurements revealed basilar invagination in 11% of patients (*n* = 8) and platybasia in 1% (*n* = 1). On presentation, patients had a median mMC score of 1 (IQR: 1–1). The CSI was 1 in 35%, 2 in 37%, and 3 in 28% of the patients (Table 1).

## Surgical management and complications

Posterior fossa decompression surgery was performed in all patients, with the absolute majority also receiving a duraplasty (96%). The remaining 4% (*n* = 3) underwent bony decompression without durotomy. Additionally, tonsillar cauterization was documented in 52% of the patients. Surgical complications occurred in 13 patients (18%). Postoperatively, CSF leak occurred in 4 patients, iatrogenic meningitis in 3 patients, subdural hematomas in 2 patients, worsening hydrocephalus in 2 patients, and a subdural hygroma in 1 patient. The majority of complications were classified as Ibanez grade 1 (Table 1). The median length of hospital stay was 6 days (IQR: 5–8). Reoperation was required in nine of the patients (13%; Table 1). Repeat PFD surgery following failure of the index procedure was performed in 7 patients, while 2 other patients had reoperations for hematoma evacuation (*n* = 1) and VP-shunt placements (*n* = 2). One patient with hydrocephalus, received an EVT prior to the Chiari decompression surgery.

## Clinical outcomes

Clinical and neurologic function were obtained at both early postoperative (median: 2 months; IQR: 1–3) and long-term follow-ups (median: 98 months; IQR: 74–122). On paired testing, posterior fossa decompression was associated with a significant improvement in mMCs (*p* = 0.003), from pre- to postoperatively at both early and long-term follow-ups. At early follow-up, 94% of patients had a CCOS ≥ 13 (improved and very good outcomes), while 6% had a CCOS between 9 and 12 (unchanged and functional outcomes). There were no patients with a CCOS ≤ 8 (worse and unfavorable outcomes). At long-term follow-up these numbers were slightly improved to 96% (CCOS ≥ 13) and 4% (CCOS 9–12). In addition, the median CCOS improved significantly between early and long-term follow-ups (*p* = 0.001). As a result, 72% of patients reached the maximum possible CCOS score of 16 at long-term, compared to 40% at early follow-up. These improvements were mainly attributable to improvements in pain (*p* = 0.044) and non-pain components of the CCOS (*p* = 0.012), rather than functionality (*p* = 0.414) and complications (*p* > 0.999) components which remained stable (Table 2).

**Table 2:** Early and long-term postoperative clinical outcomes

Follow-up (*n* = patients with available data)	Preoperatively (*n* = 71)	Early follow-up (*n* = 71)	Last follow-up (*n* = 71)	*p*-value
Clinical FU (mos)	-	2 (2)	98 (48)	-
mMC	1 (0; CI: 1.1–1.3)	1 (0; CI: 1.0–1.1)	1 (0; CI: 1.0–1.1)	0.003*
CCOS				0.927
13–16	-	67 (94%)	68 (96%)	-
9–12	-	4 (6%)	3 (4%)	-
4–8	-	0 (0%)	0 (0%)	-
CCOS (median)	-	15 (1; CI: 14.8–15.3)	16 (1; CI: 15.1–15.7)	**0.001**^**†**^
Pain	-	4 (1; CI: 3.4–3.7)	4 (0; CI: 3.6–3.9)	**0.044**^**†**^
Non-pain	-	4 (0; CI: 3.5–3.8)	4 (0; CI: 3.8–4.0)	**0.012**^**†**^
Functionality	-	4 (0; CI: 3.9–4.0)	4 (0; CI: 3.9–4.0)	0.414^**†**^
Complications	-	4 (0; CI: 3.8–4.0)	4 (0; CI: 3.8–4.0)	> 0.999^**†**^

*Paired testing considering preoperative values as reference. †Paired testing considering early follow-up values as reference. **Bold** indicates statistically significant differences. Abbreviations: CI = 95% confidence interval

### Radiological outcomes

Early postoperative and last follow-up imaging were performed at a median of 5 months (IQR: 2–8) and 61 months (IQR: 34–88), respectively for the 53 (75%) patients where complete imaging data was available. At early follow-up, the tonsillar herniation was reduced in 58%, while this number increased to 77% at last follow-up. Of the patients initially presenting with syringomyelia, 62% vs. 82% had improved, 29% vs. 14% remained unchanged, and 9% vs. 4% worsened at early vs. last follow-up, respectively. 12% of patients (*n* = 3) initially free of syringomyelia had later developed syringomyelia at last follow-up. This was due to postoperative hematoma in one case and insufficient decompression in another, both confirmed on postoperative imaging, while in the third case no apparent cause for the syringomyelia development could be identified. Of the patients initially presenting with hydrocephalus 50% improved at early follow-up. At last follow-up, hydrocephalus had improved in all patients, as those who failed to initially improve underwent CSF diversion procedures, in the form of VP-shunt placement. During the immediate postoperative period, one patient developed a subdural hygroma accompanied with transient hydrocephalus (2%), which did not require any form of treatment (Table 3).

**Table 3:** Early and long-term postoperative tonsillar, syrinx, and hydrocephalus status

Follow-up (*n* = patients with data)	Early follow-up* (*n* = 53)	Last follow-up* (*n* = 53)
**Radiological FU (mos)**	5 (6)	61 (54)
**Tonsillar status**		
Improved	31 (58%)	41 (77%)
Complete improvement	29 (55%)	15 (28%)
Partial improvement	2 (4%)	26 (49%)
Unchanged	20 (34%)	11 (21%)
Worse	2 (4%)	1 (2%)
**Syrinx status**		
Patients with syringomyelia preoperatively	34 (64%)	28 (53%)
Improved	21 (62%)	23 (82%)
Complete improvement	2 (6%)	2 (7%)
Partial improvement	19 (56%)	21 (75%)
Unchanged	10 (29%)	4 (14%)
Worse	3 (9%)	1 (4%)
No preoperative syringomyelia	19 (36%)	25 (47%)
New onset postoperative syringomyelia	2 (11%)	3 (12%)
**Hydrocephalus status**		
Patients with hydrocephalus preoperatively	4 (8%)	3 (6%)
Improved	2 (50%)	3 (100%)
Unchanged	0 (0%)	0 (0%)
Worse	2 (50%)	0/3 (0%)
No preoperative hydrocephalus	49 (92%)	50 (94%)
New onset postoperative hydrocephalus	1 (2%)	0 (0%)

*Patient groups with available early and late follow-up imaging were not overlapping

### Predictors of long-term outcomes

#### Based on pre- and perioperative parameters

A univariable proportional odds logistic regression was performed and the variables with a p-value below 0.1 were subsequently included in a multivariable proportional odds logistic regression model for the prediction of long-term CCOS (Table 4). Only motor deficits (OR: 0.09; CI95%: [0.01–0.62]; *p* = 0.015), syringomyelia (OR: 4.42; CI95%: [1.02–19.35]; *p* = 0.048), and perioperative complications (OR: 0.16; CI95%: [0.41–0.66]; *p* = 0.011) remained as significant and independent predictors of outcome. The presence of motor deficits and occurrence of perioperative complications was associated with worse CCOS, while the presence of syringomyelia was associated with improved CCOS at the last follow-up (Table 4).

**Table 4:** Proportional odds logistic regression predicting outcome (CCOS), based on pre- and perioperative parameters

Variable	Univariable *p*-value	Multivariable OR [95% CI]	Multivariable *p*-value
Older age	0.645	–	–
Female sex	0.658	–	–
Higher preoperative mMCs	0.269	–	–
Chiari severity index (CSI)	0.835	–	–
Symptoms on presentation			
Headache	0.718	–	–
Neck pain	0.763	–	–
Motor deficit	0.099*	0.09 [0.01–0.62]	**0.015**
Sensory disturbances	0.140	–	–
Sleep apnea	0.561	–	–
Dysphagia	0.426	–	–
Vertigo and impaired balance	0.089*	3.80 [0.44–33.01]	0.226
Nausea/vomiting	0.631	–	–
Radiologic findings			
Higher grade of cerebellar descent	0.123	–	–
Platybasia	0.143	–	–
Basilar invagination	0.032*	0.27 [0.57–1.30]	0.103
Syringomyelia	0.087*	4.42 [1.02–19.35]	**0.048**
Hydrocephalus	0.264	–	–
Scoliosis	0.301	–	–
Surgical approach			
Duraplasty	0.999	–	–
Tonsillar cauterization	0.671	–	–
Perioperative complication	< 0.001*	0.16 [0.41–0.66]	**0.011**

An asterisk (*) indicates a p-value < 0.1 and inclusion in the multivariable regression model. Bold text indicates a statistically significant correlation (*p* < 0.05)

### Based on findings at early follow-up

On univariable proportional odds logistic regression, neither the early postoperative tonsillar nor the early postoperative syrinx status were significant predictors of the long-term CCOS. However, the early postoperative hydrocephalus status was a significant predictor of long-term CCOS. A hydrocephalus not responding to decompression surgery during the early postoperative period (median: 5 months) was a strong predictor of worse long-term CCOS, as compared to patients with no, or those with postoperatively improved hydrocephalus (OR: 0.026; CI95%: [0.002–0.328]; *p* = 0.005). A multivariable model could not be established (Table 5).

**Table 5:** Proportional odds logistic regression predicting outcome (CCOS), based on findings at early follow-up

Variable	Univariable OR [95% CI]	*p*-value
Early tonsillar status		
Improved*	–	–
Unchanged	0.82 [0.25–2.63]	0.733
Worsened	0.079 [0.001–4.200]	0.209
Early syrinx status		
No syrinx pre- or post-operatively*	–	–
Improved	3.16 [0.77–12.93]	0.109
Unchanged	2.32 [0.48–11.15]	0.293
Worsened	0.56 [0.06–5.15]	0.609
Early hydrocephalus status		
No or postoperatively improved hydrocephalus*	–	–
Worsened	0.026 [0.002–0.328]	**0.005**

*Reference, Bold text indicates a statistically significant correlation (*p* < 0.05). N/a: not applicable

## Discussion

In this population-based retrospective study on consecutive pediatric patients treated with posterior fossa decompression for Chiari 1, favorable outcomes were achieved in the absolute majority and sustained at long term follow up. At the final follow ups, 72% had reached the maximal CCOS score of 16. This was paralleled by a gradual improvement of radiologic parameters with the proportion of patients showing a reduced grade of tonsillar herniation increasing from 58 to 77% between early and final follow-up. Similarly, the proportion of patients with reduced syringomyelia improved from 62 to 82%. Thus, the surgical decompression of the posterior fossa in Chiari 1 results not only in clinical and radiologic improvements seen in the early postoperative phase in most patients, but also translates into a gradual resolution of symptoms in the absolute majority of patients treated surgically.

From the parameters obtained in the pre- and perioperative period, we established that the presence of motor deficits and perioperative complications were significant and independent predictors of worse outcomes, while the presence of syringomyelia was a significant and independent predictor of improved outcomes. Changes in degree of tonsillar descent or syrinx at early follow up were not significant predictors of long-term outcomes. Since most patients had improved at last follow-up, this finding indicates that the lack of early radiological improvements does not indicate failure of the procedure. In contrast, we found that a hydrocephalus not responding to surgery during the early postoperative period was a strong predictor of worse long-term outcomes (*p* = 0.005). The presence of hydrocephalus alone was not predictive of worse outcomes at long-term (*p* = 0.264), but a subset of patients with hydrocephalus refractory to posterior fossa decompression did present significantly worse outcomes at last follow-up (*p* = 0.005). In a study by Yates et al., an age below 6 was associated with poor outcomes [52]. This finding could not be confirmed in this study.

The occurrence of peri- and postoperative complications were found to be a significant predictor of negative outcomes. Complications were documented in 18% (*n* = 13) of the cohort, with only 15% (*n* = 2) of the complications requiring a procedure under general anesthesia. Thus, the documented complications mainly reflect the most common and largely inconsequential occurrences associated with any surgery. However, the panorama in Chiari 1 also includes severe complications such as meningitis, posterior fossa hematomas, subdural hygromas or hematomas and CSF-leakage. These complications may have a profound long-term impact on the patients’ health related quality of life, which is reflected in the complications dimension of CCOS.

Studies on prognostic factors in pediatric Chiari 1 have resulted in tools such as the CSI and CCOS [16, 24, 33, 43, 48, 54]. CSI has also been used as an outcome predictor in adult populations [19, 33]. However, subsequent validation studies in pediatric populations have been unsuccessful [2, 20]. This study agrees with previous studies on pediatric populations, failing to demonstrate CSI as an outcome predictor (p.a. = 0.835).

The literature on adult Chiari 1 point to a negative impact of syringomyelia on clinical outcomes [1, 12, 16, 37]. However, there are studies, including a recent meta-analysis, that fail to identify syringomyelia as a significant outcome predictor [8, 17, 20]. Conversely, our study and a study by Hekman et al. [24], mainly addressing pediatric populations, find syringomyelia an independent and positive predictor of outcomes. However, the early postoperative syrinx status (at a median of 5 months postoperatively) did not predict long-term surgical outcomes, which corroborates findings from previous studies [28, 48]. To accommodate these somewhat diverging data, syringomyelia must be identified as a marker of symptomatic Chiari-I, reflecting obstruction of normal CSF flow in and around the cervical medulla at the level of the obex [26]. However, syringomyelia is not itself the cause of typical symptoms such as occipital headaches or vertigo. Ideally, syringomyelia resolves once the obstruction to CSF flow is removed. However, long-standing distention of the central canal may be slow to recover which partly explains the poor utility of postoperative syrinx-status in predicting outcomes. Noteworthy, syringomyelia has also been reported to occur secondarily to membranous obstruction at the obex, a condition that is found in close to 50% of pediatric patients with Chiari type 1 [29]. The condition is underdiagnosed and undertreated as it is not readily visualized on MRI and requires the surgeon to open the dura and perform a microdissection to provide a clear view of the obex, after which the membrane can be opened. In these cases, posterior fossa decompression without fenestration of the obstructing membrane may manage typical Chiari I symptoms without relieving the syringomyelia. This is corroborated by several previous studies. Ene et al., in a series of 276 pediatric patients with Chiari 1, found that patients presenting with a syrinx who underwent expansile duraplasty with obex exploration, had a significantly greater likelihood of syrinx and symptom resolution, without increased risk of related complications, as compared to those who underwent only bony decompression [15]. Another study on 82 patients, indicated that unblocking the obex resulted in better syringomyelia resolution compared with duraplasty alone or bony decompression alone without increased postoperative complications or worse clinical outcomes [23]. In a study by Kleklamp et al., on 133 patients, the authors recommended that surgery should consist of a small craniectomy, opening of the dura, and arachnoid dissection to re-establish normal cerebrospinal fluid outflow from the 4th ventricle [31].

The current study revealed that the early hydrocephalus status, at a median of 5 months postoperatively, was a significant predictor of outcomes. In fact, patients with new onset postoperative hydrocephalus or those with worsening hydrocephalus were more likely to present worse CCOS scores at last follow-up.

Similarly, hydrocephalus was one of the most important variables contributing to a recently developed machine learning model that was able to predict reoperation and complication in pediatric patients undergoing PFD for CM1, with high accuracy [13]. Also, in the study by Greenberg et al. hydrocephalus was the strongest predictor of postoperative complications [21]. Conversely, in a study by Vedantam et al., hydrocephalus was associated with perioperative adverse events on univariable analysis but did not maintain statistical significance on multivariable analysis [49]. 

Hydrocephalus may arise secondary to Chiari type 1 but may also be preexisting in which case the Chiari 1 is unrelated or even secondary to the hydrocephalus itself. In cases where the Chiari 1 malformation causes hydrocephalus, persistent or worsening of the hydrocephalus may reflect inadequate decompression of the posterior fossa. Inversely, in cases where tonsillar descent occurs as a result of an underlying hydrocephalus, surgery with posterior fossa decompression may paradoxically result in postoperative clinical decline due to increased herniation through the foramen magnum [45]. Hence, the establishment of strategies to effectively diagnose and manage hydrocephalus is pivotal in improving long-term outcomes after posterior fossa decompression in children with Chiari type 1 [14, 22, 38, 45]. 

The predictive value of preoperative radiographic measurements has been studied previously, with CTD, platybasia, and basilar invagination being the most studied parameters [8]. Liu et al., found all three parameters to be significant predictors of poor outcomes [36]. In line with this, Yilmaz et al. reported that a low CTD grade was a predictor of favorable postoperative outcomes [53]. Basilar invagination has similarly been reported to be associated with poor outcomes [7, 20]. In this material, only basilar invagination was associated with worse outcomes on univariable regression, but the significance was not maintained on multivariable regression. In fact, a recent meta-analysis, although limited by a small sample of included studies, came to the conclusion that there was no significant association between basilar invagination and postoperative outcomes [8]. 

### Strengths and limitations

One of the main strengths of this study is its population-based nature, as the study center acts as the only neurosurgical center for the surrounding region of 2.3 million inhabitants. Moreover, the study provides exceptionally long-term postoperative clinical and radiologic outcomes with follow-ups extending a median of 8 and 5 years, respectively. The findings reported in this study reflect a single center material, which may introduce biases related to clinical decision making. Thus, similar studies performed at other institutions are needed to confirm generalizability of the findings and conclusions. Finally, a small proportion of patients were lost to imaging follow-up.

## Conclusion

In conclusion, surgical treatment of Chiari 1 in pediatric patients leads to favorable outcomes that may improve over time. Surgery leads to immediate and sustained improvements regarding the tonsillar, syrinx, and hydrocephalus status. Results from this study indicate that pre- and early postoperative parameters were effective at predicting long-term outcomes. The presence of motor deficits, the occurrence of perioperative complications, and the absence of syringomyelia were all significant and independent predictors of worse outcomes measured by CCOS. A hydrocephalus not responsive to the posterior fossa decompression was predictive of worse long-term CCOS scores. Implementation of strategies for the prevention of perioperative complications and appropriate management of hydrocephalus is essential to achieve good outcomes following posterior fossa decompression in Chiari 1 patients.

## Data Availability

Deidentified data may be provided upon reasonable request directly addressed to the corresponding author.
